# Low-Cost Wireless Structural Health Monitoring of Bridges

**DOI:** 10.3390/s22155725

**Published:** 2022-07-30

**Authors:** Seyedmilad Komarizadehasl, Fidel Lozano, Jose Antonio Lozano-Galant, Gonzalo Ramos, Jose Turmo

**Affiliations:** 1 Department of Civil and Environment Engineering, Universitat Politècnica de Catalunya, BarcelonaTech. C/Jordi Girona 1-3, 08034 Barcelona, Spain; milad.komary@upc.edu (S.K.); gonzalo.ramos@upc.edu (G.R.); 2 Department of Civil Engineering, Universidad de Castilla-La Mancha, Av. Camilo Jose Cela s/n, 13071 Ciudad Real, Spain; fidel.lozanogalant@uclm.es (F.L.); joseantonio.lozano@uclm.es (J.A.L.-G.)

**Keywords:** Arduino Due, Raspberry Pi, accelerometers, low-cost sensors, eigenfrequency analysis, short-span footbridge

## Abstract

Nowadays, low-cost accelerometers are getting more attention from civil engineers to make Structural Health Monitoring (SHM) applications affordable and applicable to a broader range of structures. The present accelerometers based on Arduino or Raspberry Pi technologies in the literature share some of the following drawbacks: (1) high Noise Density (ND), (2) low sampling frequency, (3) not having the Internet’s timestamp with microsecond resolution, (4) not being used in experimental eigenfrequency analysis of a flexible and a less-flexible bridge, and (5) synchronization issues. To solve these problems, a new low-cost triaxial accelerometer based on Arduino technology is presented in this work (Low-cost Adaptable Reliable Accelerometer—LARA). Laboratory test results show that LARA has a ND of 51 µg/√Hz, and a frequency sampling speed of 333 Hz. In addition, LARA has been applied to the eigenfrequency analysis of a short-span footbridge and its results are compared with those of a high-precision commercial sensor.

## 1. Introduction

Civil infrastructures and structures might be considered the leading basis of modern society; consequently, their health state is of the highest importance [1]. However, data provided by the American Society of Civil Engineers (ASCE) infrastructure indicates about 9.1% of bridges in the United States of America are structurally inefficient. Every day, over 188 million trips are taken across structurally deficient bridges. In addition, in average, a bridge in the United States of America is 43 years old, which is close to its expected life span [2]. 

Evaluating these structures along with their health-state assessment is needed to reduce reparation costs, maintenance, and, eventually, for certifying infrastructure/structure safety [3,4,5]. Structural Health Monitoring (SHM) applications issue statistics on structures’ functioning states and their structural responses [6,7,8].

For measuring the structural responses (such as deflections, strains, rotation, temperature, humidity, and accelerations) over time, sensors are widely used in SHM systems [9,10]. The recorded information of the sensors is then used for structural performance estimations [11,12,13]. In fact, natural phenomena can be categorized as static or quasi-static and dynamic [14,15,16]. On the one hand, some environmental elements (such as temperature or humidity) change very slowly, so they can be considered to perform either as quasi-static or static [17,18]. On the contrary, some events (such as traffic-induced vibrations, ambient activities, and waves from seismic activities) are considered dynamic because their changing rate is significantly affected by the time [19,20,21].

Accelerometers are force-sensors that are broadly used for measuring vibrations [22]. According to [23], accelerometers are frequently set up on one of the following principles:

(1) Piezoelectricity: This is one of the most popular types of accelerometers in the industry for measuring [24], based on specific materials’ piezoelectric effect. For measuring the dynamic changes in mechanical variables, the piezoelectric effect of particular materials is used [25]. The main advantage of these devices is that they can operate on a wide range of frequencies (up to 12 kHz) [26];
(2) Piezoresistivity: The second most popular vibration acquisition sensors are piezoresistive (strain gauge) accelerometers. This kind of accelerometer measures the change in electrical resistance of a particular piezoresistive element when vibrations are induced [23];
(3) Differential capacitive measurement: These accelerometers identify vibrations by measuring the capacitance changes of a seismic mass [23];
(4) Micro-Electro-Mechanical Systems (MEMS) are silicon-based microsensors built from any principles mentioned above [23].

It is essential to mention that the use of accelerometers varies in different SHM applications [27,28]. According to the literature (see, e.g., [29]), most civil structures’ significant natural frequencies vary between 0.2 and 100.0 Hz. The eigenfrequency range of short-span bridges (up to 40 m of span length [30]) usually ranges between 3 and 30 Hz [31,32,33]. Medium- and long-span bridges have eigenfrequencies ranging between 0.1 and 8.0 Hz (such as [34,35,36]). It should also be noted that most of the ambient vibrations in civil structures are characterized by low amplitudes [37,38]. In fact, the acceleration amplitude of these structures can be as low as 0.04 g. This characteristic shows the necessity of using accelerometers with low noise density and high sensitivity for SHM of bridges [39]. The commercial MEMS accelerometers have a bandwidth of a few kHz. However, their design for higher frequencies has a lower noise density [23,40]. Finally, it should be highlighted that the sensitivity of an accelerometer has an indirect ratio with the squared value of the resonant frequency. For that reason, the higher the accelerometer’s sampling frequency, the lower its sensitivity [41]. For that, many researchers tried to develop low-cost MEMS accelerometers with limited sampling frequency and vibration amplitude range for civil SHM [42].

A literature review on various low-cost MEMS accelerometers is organized in the following paragraphs. Even though many applications use low-cost accelerometers, only a handful of them were used in civil engineering.

1. Grimmelsman et al. [43] studied a low-cost accelerometer (ADXL335). In their work, this low-cost accelerometer’s performance and capability were compared with conventional instrument-grade accelerometers (PCB 393A03 and 3741E122G);
2. For providing the structural modal analysis from several synchronized low-cost accelerometers (LIS344ALH) distributed along with a structure, a system was proposed by Girolami et al. [44];
3. The research of Ozdagli et al. [45] used the accelerometer MPU6050 for a real-time, Low-cost, Efficient Wireless Intelligent Sensor (LEWIS). In the performed experiments, LEWIS was placed on a breadboard for signal acquisition. Moreover, its measurements were compared with a Linear Variable Differential Transformer (LDVT) sensor and a commercial accelerometer (PCB 3711B1110G);
4. An updated version of LEWIS is proposed by Aguero et al. [46]. This work describes LEWIS 2, which solved three main drawbacks of the first LEWIS. It is mentioned in the introduction that LEWIS 2 has data storage, is battery-powered, and is more accurate than LEWIS. The measurements were compared to those of a LDVT sensor;
5. Meng et al. [47] propose a low-cost acquisition system composed of a LSM9DS1 accelerometer and a Raspberry Pi 4. This system was evaluated with a commercial accelerometer (PCB 356B18);
6. Recently a Cost Hyper Efficient Arduino Product (CHEAP) was introduced by Komarizadehasl et al. [23]. Different laboratory tests were performed to validate CHEAP’s performance, and the results of CHEAP were compared with those of two commercial seismic accelerometers (PCB 393A03 and PCB 356B18).

Table 1 summarizes the essential characteristics of the reviewed accelerometers. It includes the following information collected in columns (1) sensor number, (2) the name of the accelerometer, (3) acceleration range: the maximum acceleration amplitude capacity of the sensors, (4) sampling frequency: the sampling speed rate of the accelerometer—also, half of this amount corresponds to the accelerometer’s bandwidth, (5) spectral Noise Density (ND), (6) NTP: the capability of the sensor’s clock for getting constantly synchronized with the exact time of the Internet, and (7) Internet access: being connected to the Internet for data uploading or wireless access.

**Table 1:** Low-cost solutions in the literature to measure accelerations.

No.	Accelerometer	Acceleration Range (g)	Sampling Frequency (Hz)	Spectral Noise (µg/√Hz)	NTP	Internet Access
1 [43,48]	ADXL335	±3 g	100	300	No	No
2 [44,49]	LIS344ALH	±2 g	100	50	No	Yes
3 [45,50]	MPU6050	±2 g	100	400	No	No
4 [46,51]	MPU9250	±2 g	500	300	No	No
5 [47,52]	LSM9DS1	±2 g	952	No data	Yes	Yes
6 [23]	CHEAP	±2 g	85	No data	No	No

The analysis of Table 1 shows that almost all the systems have more or less the same acceleration range and sampling frequency. LSM9DS1 (No. 5) is the only accelerometer with a very high sampling frequency that needs further investigation. Furthermore, the shown ND in Table 1 is reported from the datasheets provided by the sensor producer company. This has been calculated in a standard temperature (25 degrees Celsius for MPU6050) and perfect laboratory conditions. The presented low-cost solutions of Table 1 have not tested the ND of their representatives after they have wired them up. The device of Meng et al. [47] is the only intelligent system with the ability to synchronize its internal clock with the exact time of the Internet through the NTP procedure. The analysis of Table 1 further shows that only sensors numbered 2 and 5 can access the Internet for uploading their results in a cloud server.

Further study of the information as mentioned earlier presented in the literature review shows a lack of consistent work, including all the following points in single inception for a low-cost accelerometer: (1) access to the time through Internet, for accurate sensor synchronization, (2) wireless control, (3) scheduling for a synchronized data acquisition experiment, and, finally, (4) measuring the ND and data resolution after the system was wired and (5) performing experimental eigenfrequency analyses on actual infrastructures.

This paper aims to adapt CHEAP to solve all the detected problems and to develop a new Low-cost Adaptable Reliable Accelerometer (LARA). LARA has the following main improvements: (1) higher bandwidth: achieving a faster sampling frequency (333 Hz) by efficiently rewriting all the library codes, (2) independency: this accelerometer is not dependent on any connected computer for data acquisition—LARA has an attached low-cost microprocessor that allows the acquired data to be saved on an SD card or any portable flash or hard drive, (3) triaxial data acquisition: having all the accelerometers of LARA in *x*, *y,* and *z* direction aligned makes the final product suitable for triaxial data acquisition—besides, if needed, it can work as a uniaxial, biaxial accelerometer, (4) low Noise Density, (5) Internet timestamp and synchronization: the Network Time Protocol (NTP) of LARA is activated. Firstly, NTP synchronizes the clock of each LARA with the accurate time of the Internet constantly. Secondly, each acquired vibration receives an accurate timestamp. This is the feature that makes post-processing of sensor synchronization possible. The literature review reveals no other proper sensor synchronization solution that uses free software. Instead of having Data Acquisition (DAQ) system, LARA uses python software for scheduling a measurement. This way, different LARAs can start acquiring information simultaneously in other parts of a structure, (6) experimental analysis: in this paper, a short-span was instrumented and the eigenvalue analyses of LALA are compared with those of certified vibration sensing devices, and (7) wireless accessibility: the user can have access to the saved data wirelessly through the Internet. Therefore, LARA can be controlled, get upgraded through online commands and data can be recorded. In addition, LARA’s weight is around 207 g which makes it 150 g or 58% lighter than CHEAP.

A laboratory test campaign was designed to validate LARA’s performance against its older version. In this set of experiments, frequencies ranging from 0.1 Hz to 32 Hz and acceleration amplitudes in the range of [0.001–9.806 m/s^2^] were tested.

Furthermore, in this paper, another field test is performed on a short-span footbridge in Barcelona. In this experiment, the eigenfrequencies of LARA are compared with a commercial dynamic sensor (HI-INC) [53]. HI-INC is a dynamic inclinometer with a sampling frequency of 250 Hz. This sampling frequency is enough for acquiring the main mode shapes of the footbridge under study. It should be noted that the reported noise density of the used commercial sensor (HI-INC) in its datasheet is 0.0004 degrees/√Hz. As in other works in the literature (see, e.g., [54]), this MEMS inclinometer was only used for comparing the eigenfrequency analysis of LARA.

This paper is organized as follows: in Section 2, LARA (the updated version proposed by this paper) is presented with its detailed characteristics. In Section 3, the validating laboratory tests of CHEAP, LARA, and MPU9250 are presented together with their results. In Section 4, the eigenfrequency validation of LARA on a short-span bridge is presented. Finally, the main conclusions are drawn in Section 5.

## 2. Triaxle Wireless Low-Cost Adaptable Reliable Accelerometer (LARA) with Post-Synchronization Capability

In this section, the main features and innovations of the Low-cost Adaptable Reliable Accelerometer (LARA) in terms of software and hardware are detailed.

LARA is an updated CHEAP version, a wireless triaxial accelerometer that can be controlled, monitored, and programmed wirelessly.

**Figure 1:**
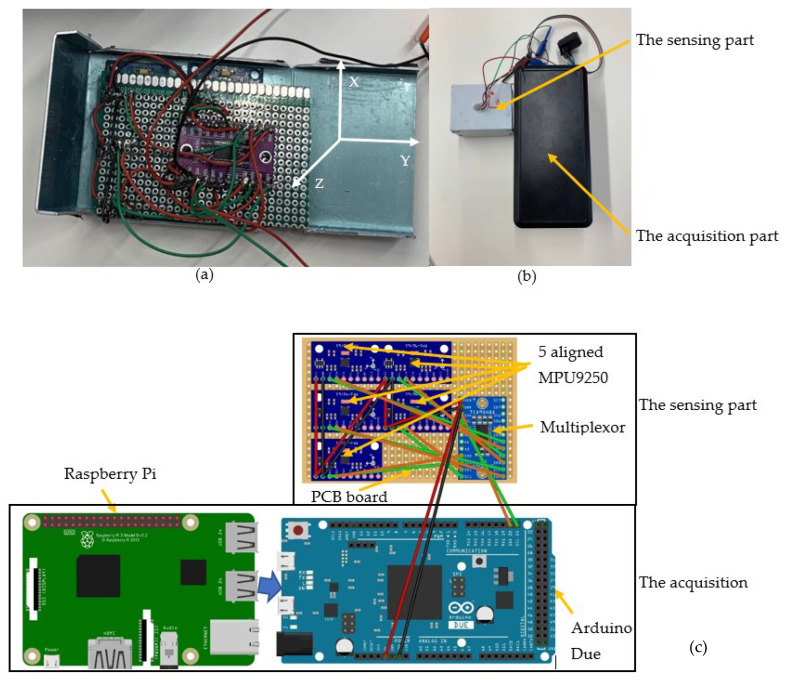
LARA elements: (**a**) the adjustments and wire connections of the sensing part, (**b**) the sensing and acquisition part, and (**c**) LARA in detail.

This new accelerometer has a frequency sampling of 333 Hz, an acceleration range of ±2.0 g, and effective bandwidth of 165 Hz. LARA is built into two parts: (1) A sensing part: this contains the aligned accelerometers and the multiplexor, which is shown in Figure 1a. It should be noted that, the same as CHEAP, LARA consists of five aligned low-cost MEMS accelerometers. It is due to the fact that when the results of a few accelerometers with unique inherent dynamic noises are averaged, the signal under study remains invariable. However, the dynamic noises of individual sensors are divided by the square root of the number of averaged sensors, and (2) an acquisition part: consisting of an Arduino and a Raspberry Pi. The sensing and acquisition parts of LARA are shown in Figure 1b. In addition to the CHEAP components, LARA includes:

A Raspberry Pi: Raspberry Pi is a small size Linux-based computer that can be connected to Arduino microcontrollers. This way, the operator can access the Arduino codes for modifying or upgrading purposes. To save and acquire provided data of the accelerometers, a python code was written to save the acquisition data on the Raspberry Pi memory card. For synchronizing different LARAs for future SHM applications, the Arduino’s acquired data were saved with an accurate timestamp. The timestamp is reported from the inner clock of the Raspberry Pi. For constantly synchronizing the inner clock of the Raspberry Pi with the accurate time of the Internet, the Raspberry Pi’s NTP protocol was activated. Figure 1c shows the connection between the Arduino and the Raspberry Pi. Arduino gets its needed power from a USB port of the Raspberry Pi. Raspberry Pi can be powered up by using an adaptor or a power bank. It is also noteworthy to mention that the time keeping accuracy when using a Raspberry Pi model 3b+ with a NTP server was already published in the literature [48]. In this work, an overall deviation of 0.01 s was measured after a 40 h test. This value corresponds to a time accuracy of 0.07 Parts Per Million (PPM).

A PCB board: a Printed Circuit Board (PCB) was used to place the sensors in the targeted location with all their axes aligned. The sensors used the shortest possible wire lengths for connections between the multiplexor the accelerometers. In Figure 1a, the adjustment of sensors on LARA by the PCB is shown. In fact, it can be seen in Figure 1a,c that the PCB board has aligned *x*, *y,* and *z* axes of the MPU9250 accelerometers.

An aluminum box: the sensors are placed inside a box to preserve the accelerometers from environmental conditions (such as humidity, dust, and environmental activities). A very rigid and stiff material was needed to reach the same input signal to all the accelerometers [55]. Aluminum material was chosen to hold the accelerometers because it is very stiff, but, at the same time, it is a very light and conductive material. The conductivity helps the sensor’s grounding. Besides, in Figure 1b, the boxing of LARA is illustrated. The dimensions of this element are 52 × 72 × 44 mm.

A USB 4G dongle: this device included a modem with a USB dongle with 4G Internet connection connected to the Raspberry Pi that was used for the following purposes: (1) providing the accurate time of the Internet for the data acquisition timestamp, (2) controlling remotely the data acquisition process, and (3) transferring wirelessly the acquired data from the memory card of the Raspberry Pi to another computer.

A plastic box for the acquisition equipment: a plastic box was used to preserve the Arduino, Raspberry Pi part, and the Internet dongle. The connection between two boxes is within four sets of wires. The red, black, green, and orange wires connect the 5-volt Voltage Common Controller (VCC), GrouND (GND), Serial Clock Line (SCL), and Serial Data Line (SDA) of the sensing part to the acquisition part.

Besides the extra hardware parts that LARA possesses compared with CHEAP, LARA uses a new code and a library code that makes it faster and triaxial. The software enhancement of LARA refers to:

(1) Increasing sampling frequency: frequency sampling of LARA is increased by rewriting the old library code and using a faster communication clock along with the main code;
(2) No coding error: increasing the frequency sampling of a system can result in error reporting, interruptions, data loss, or fluctuation in the frequency sampling speed. In an experiment, LARA worked for an entire week and saved data with no errors or interruptions or data loss;
(3) Schedule data acquisition: a code has been prepared on python to schedule and end vibration acquisition. This scheduling has two benefits. Firstly, it makes wireless sensor synchronization possible with free software. Secondly, it can be used for OMA applications. It should be noticed that when a structure is heavily excited by ambient causes, the accelerometers can extract more valuable data. Therefore, an accelerometer with schedule capability can help acquire data only when the structure is under high traffic or extreme activity;
(4) Internal sensor synchronization lag enhancement: the accelerometers inside each LARA are not 100% synchronized. In fact, the Arduino executes codes one line at a time. When the main code is executed, the Arduino opens the library code and uses the information to get the first sensor’s acceleration, and after the second one, and so on. This operation takes time. In the CHEAP, the lag between each sensor-print was about 2200 microseconds. By the hardware and software improvement, the corresponding lag of LARA is decreased to 210 microseconds, which is 10.47 less than the lag of CHEAP. In fact, a lower lag time contributes to a better sensitivity of LARA for acquiring higher frequencies;
(5) Post-synchronization of several LARAs: to use LARA’s outputs in the OMA application, various accelerometers’ data must first be synchronized and have the same sampling frequency. Since LARA has access to the accurate time of the Internet, the acquired data can be stamped with the precise time of the Internet with microsecond resolution. This timestamp helps calculate the sampling frequency of each LARA precisely and, more importantly, the fluctuation of the vibration acquisition process. Even though the reported sampling frequency of each LARA is calculated by measuring the number of acquired data during the acquisition process, the fluctuation is calculated by measuring the needed time for saving 100 data. It is seen through laboratory experiments that when the input power is insufficient, the fluctuation is unsteady. This insufficient input power can be due to low speed of the used USB wire, the long length of the USB cable, the imperfection of the used power bank, or simply using a power source that cannot provide 2.5 A and 5 V [9].

## 3. Laboratory Tests and Results

This section aims to validate the performance of LARA. Firstly, the resolution, sensitivity, and ND of a single MPU9250, CHEAP, and LARA are presented. For that, an extended duration test was performed in an office. Secondly, a test has been carried out on the hydraulic jack of the laboratory. In that test, LARA was validated and compared with CHEAP. Next, the performed tests for validating LARA’s accuracy in terms of various frequencies are investigated. Finally, the acceleration amplitude evaluation is shown.

It should be noted that the performance of CHEAP was validated using two high-precision accelerometers (PCB 393A03 and 356B18) [23] in several laboratory experiments for a range of frequencies from 0.5 to 10 Hz. These tests were performed on the same shaking platform used in this work. This platform is an INSTRON 8803 model located at the Structural Laboratory Lluís Agulló of the Technical University of Catalonia (Barcelona, Spain). In addition, it is essential to mention that LARA was calibrated in the laboratory of the Applus company. In that certification, the acceleration amplitude accuracy of LARA was studied in several experiments with a fixed RMS acceleration amplitude of 0.5 g within the range of 5 to 160 Hz.

### 3.1. Sensitivity, ND, and Resolution Evaluation

This section compares the sensitivity, ND, and resolution of a single MPU9250 accelerometer with CHEAP and LARA.

The accelerometers’ sensitivity can be defined as the ratio of the input (induced vibrations of the shaking table) to the output (the information reported by the accelerometer). This concept is measured and calculated differently for analog and MEMS sensors. Although an analog accelerometer usually produces electronic pulses related to the input vibrations, MEMS accelerometers convert these electronic pulses into digital signals. While the sensitivity of analog sensors is reported as V/g (Voltage per gravitational acceleration), MEMS accelerometers report their sensitivity in the Least Significant Bit per gravitational acceleration (LSB/g) [56]. Since this value is a characteristic of a sensor, it should be the same for MPU9250, CHEAP, and LARA. The datasheet of MPU9250 reports a sensor sensitivity for the acceleration amplitude range of ±2.0 g of 16,384 LSB/g. MPU9250 accelerometers work with an operating voltage of 2.5 volts and their scale for converting data from analog to digital (Analog to Digital Converter ADC) is 16 bits [47]. The ADC formula for calculating the LSB is shown in Equation (1) [56].

(1) $$\mathrm{LSB}=\frac{\mathrm{Input}\;\mathrm{voltage}}{2^{\left(\mathrm{number}\;\mathrm{of}\;\mathrm{bits}\right)}}$$

In Equation (1), with an input voltage of 2.5 and an ADC scale of 16, each unit of LSB is equivalent to 0.03814 mv. In this way, with 16,384 LSB/g for acceleration amplitude range of ±2.0 g, the comparable sensitivity of MPU9250, CHEAP, and LARA is calculated as 625 mV/g.

To calculate ND and resolution of the devices, a long-term test was performed. The ND results of the different accelerometers are calculated using Equation (1) [57]. It should be noted that CHEAP was located to read the signals from the *Z* direction. For validating this test and the used formula, an MPU9250 accelerometer was tested. In addition, the reported results of its datasheet are compared with the results of the tests. It should be noted that the presented noise-density measurement of this work is a standard procedure to characterize the noise of the developed accelerometer [58]. This, typically, appears on the datasheet of commercial accelerometers [59]. However, it should be noted that those applications that aim to characterize the noises throughout time (such as Allan variance or Allan deviation) are out of the scope of this paper. Typically, these applications are used to investigate the noises of sensors (such as gyroscopes) which data drift throughout time in the time-domain series.

(2) $$ND=\sqrt{\frac{\sum_{1}^{N}{\left(x_{i}-\mu \right)}^{2}}{N*f}}$$

In Equation (2), the $x_{i}$ is the reported values of the accelerometers in the time-domain, *μ* is the average of all $x_{i}$ values, *N* is the number of used samples, and *f* is the sampling frequency of the accelerometer.

The calculated ND of MPU9250, CHEAP, and LARA for the *z*-directions are 390, 162, and 81 µg/√Hz, respectively. In addition, the ND of LARA for both *x* and *y* directions is 51 µg/√Hz.

The datasheet of MPU9250 reports its ND as 300 µg/√Hz. In fact, the illustrated information of datasheets is usually acquired under the best possible circumstances. It can be deducted from the calculated ND that using shorter wires and better connections made LARA 50% less noisy than CHEAP by reducing the ND value from 162 to 81 µg/√Hz in the *z*-direction. The calculated ND values also shows that LARA has almost 79% less ND on the *z* axes than a single MPU9250 accelerometer by reducing the ND value from 390 to 81 µg/√Hz. Further studying of these values shows a lower ND of LARA on the *x* and *y* axes. These two axes do not measure the gravitational acceleration of the earth; subsequently, the ND of LARA is 51 µg/√Hz.

**Figure 2:**
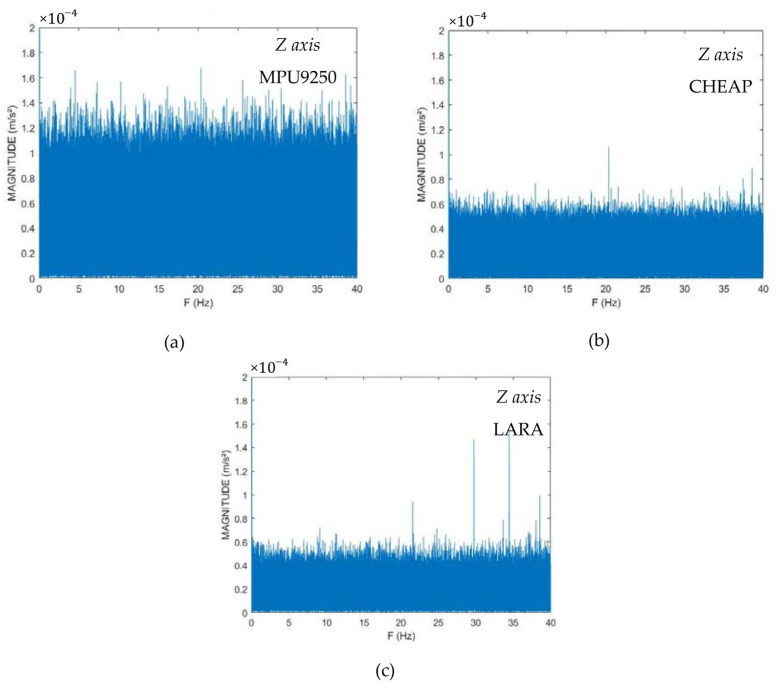
Frequency domain diagrams for *z* axis of: (**a**) MPU9250, (**b**) CHEAP, and (**c**) LARA.

Since the evaluation of the accelerometer’s resolution depends on the number of samples [60], for a fair comparison between devices with various sampling frequencies, the same number of the acquired samples were used in the test. For illustrating the resolution of the accelerometers, their reported data have been transformed from time-domain to frequency-domain by a Fast Fourier Transform (FFT). The results for different sensors are reported in Figure 2.

**Figure 3:**
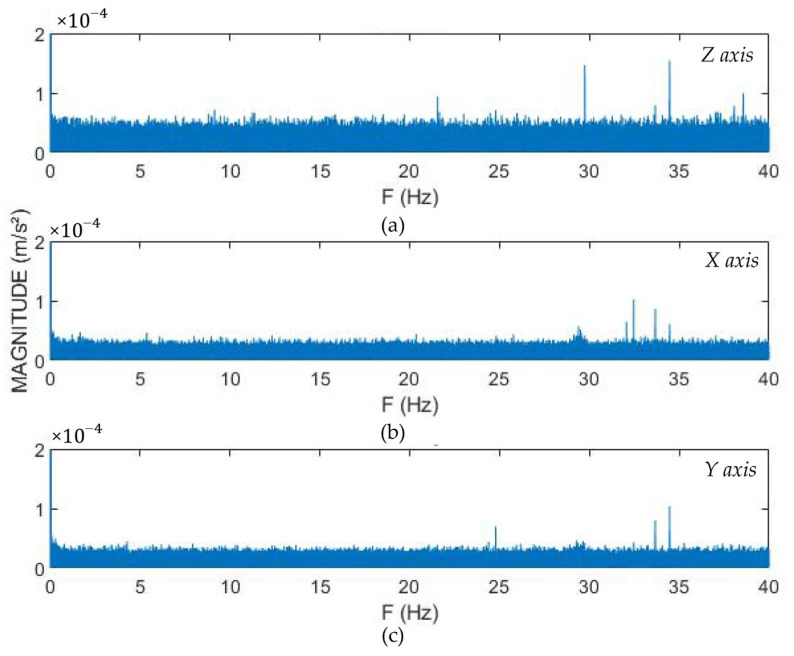
Frequency domain diagrams of LARA for: (**a**) *z*, (**b**) *x,* and (**c**) *y*-axis.

Analysis of Figure 2 shows that while LARA and CHEAP resolution are almost equal, the resolution of a single MPU9250 is almost twice as the CHEAP or LARA. By studying Figure 2, MPU 9250’s resolution, CHEAP and LARA for the z-axis are reported as 0.00016 m/s^2^, 0.00009, and 0.00009, respectively. It is to be considered that this test needed a long duration of data capture; consequently, it was done in an office at midnight. The FFT method reports more accurate outputs when it has a higher number of inputs [60]. For a fair comparison between MPU 9250, CHEAP, and LARA, the same number of data had to be evaluated by the FFT method. Although sampling two million sets of data took MPU9250 and CHEAP 6.5 h, LARA acquired two million sets of data in about 1.7 h. Figure 3 shows the frequency domain diagrams of acquired data of LARA for all the axes.

Analysis of Figure 3 shows that the resolution of LARA for *x* and *y* axes is 0.00005 m/s^2^. The resolution for *x* and *y*-directions is 44% better than the *z*-axis because of the absence of gravitational acceleration of the earth in those directions. As a result, the resolution of this accelerometer is evaluated as 0.00005 m/s^2^.

After calculating LARA’s sensitivity, ND, and resolution, LARA can be compared with instrument-graded accelerometers. In Table 2, various commercial triaxial MEMS applications are presented. The contents of this table are ordered by the noise density of the accelerometers. This table includes the following information, which is reported by the datasheets provided by the producers or measured in this paper, organized in columns: (1) sensor number, (2) sensor name, (3) acceleration range, (4) sampling frequency speed, (5) Noise Density (ND): the RMS resolution can be calculated by multiplying the ND by the square root of the sampling frequency, (6) sensitivity: for a better comparison, the analog converted sensitivities by the producer companies are reported for each product, (7) price of the sensor (VAT excluded): prices are based on the recent declaration of the producer. The reported price of LARA, CHEAP, and MPU9250 refers to research prototypes and includes the used inceptions (such as accelerometer, Arduino, wires, multiplexor, and Arduino) in them. It should be noted that the rest of the sensors are commercial solutions. The information in this column is to illustrate the price ranges and not to compare the price of the prototype sensors with the commercial ones, and (8) acquisition equipment.

Analysis of Table 2 shows that noise level of the commercial solutions (IAC-Hires [61], 3713F112G [62], Unquake [63], and Recovib Tiny [64]) ranges from 8 to 30 µg/√Hz. In addition, analyzing Table 2 indicates that LARA has a more comparable ND with the introduced commercial accelerometers in Table 2 than with any of the low-cost accelerometers (5–7) presented in Table 1. The noise level of the commercial solutions (IAC-Hires [61], 3713F112G [62], Unquake [63], and Recovib Tiny [64]) ranges from 8 to 30 µg/√Hz.

It is shown in Table 2 that 3713F112G and IAC-Hires need extra data acquisition equipment for acquiring the vibrations. In fact, 3713F112G needs a signal conditioner such as 482C27 with a price of 5070 € with four channels, and the IAC-Hires requires a data acquisition such as Recovib Monitor with a price of 3700 €, which can provide up to eight reading channels. Moreover, CHEAP and MPU9250 are dependent on an attached computer for their signal monitoring and saving.

However, Unquake, Recovib Tiny, and LARA do not need any acquisition system for their data acquisition. Accelerometer Unquake samples internally, and the measurements are timestamped with absolute time from GPS. The measurements are synchronized in the post-processing stage based on the timestamps by using the software that comes for free from the company. In addition, Recovib Tiny and LARA are both wireless sensors.

Further analysis of Table 2 shows a wide range of prices (varying between 1192 € up to 2500 €) of commercial solutions. In fact, as stated in [65], the cost of accelerometers is known to be one of the critical limitations of SHM analysis and long-term monitoring.

### 3.2. Frequency Validation

This subsection illustrates the experiments aiming at the frequency measurement accuracy of MPU9250, CHEAP, and LARA on a vibration platform.

An experiment to validate the frequency report of LARA is presented in this section. A jack that could induce displacements with a known frequency was used. Although the jack is very accurate in reproducing a specific frequency, its performance is limited in movement. Therefore, for every experiment, the jack presents a report. This report is the time-domain information of the activities of its lower jaw.

**Figure 4:**
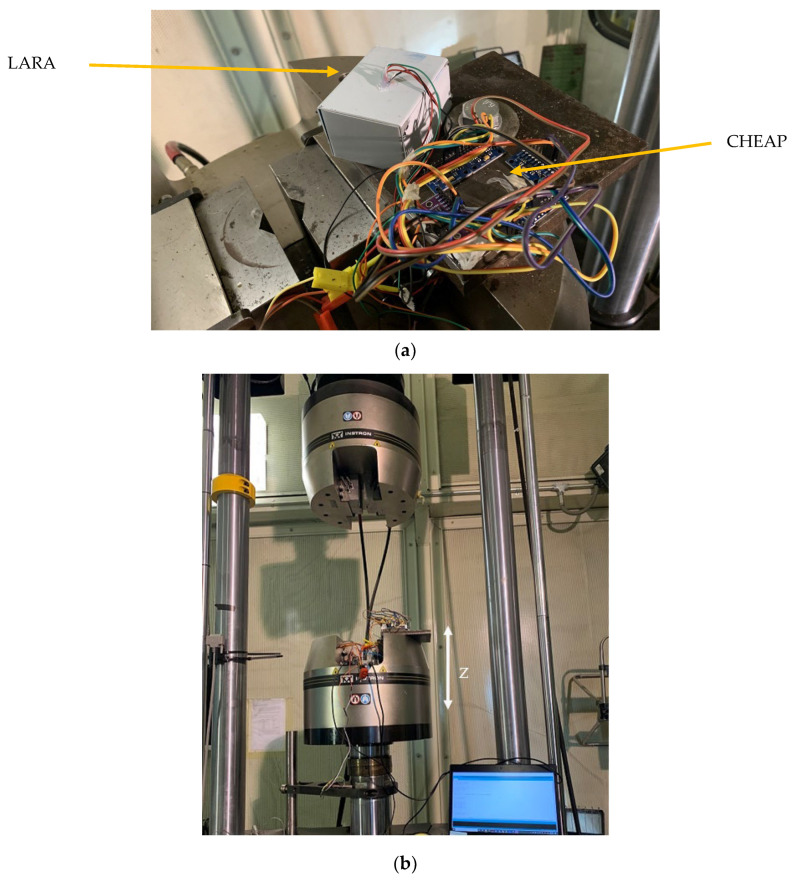
Laboratory validation of LARA: (**a**) mounting CHEAP and LARA to the shaking part of the jack, and (**b**) the used vibrating platform (INSTRON 8803).

It should be noted that the vibration platform used in this paper (INSTRON 8803) was programmed using WaveMatrix2 Dynamic Software This jack can create various waveform types with the frequency range of 0.1 to 100 Hz and its resonant frequency is 134 Hz. The movement direction of this vibration platform (*z*-direction) is shown in Figure 4b.

In this work, the waveform is set to a sine wave. All the additional technical features of this hydraulic jack, its programming software, and its datasheets are presented in [23].

Figure 4 shows the placement of the CHEAP and LARA on the hydraulic jack.

**Figure 5:**
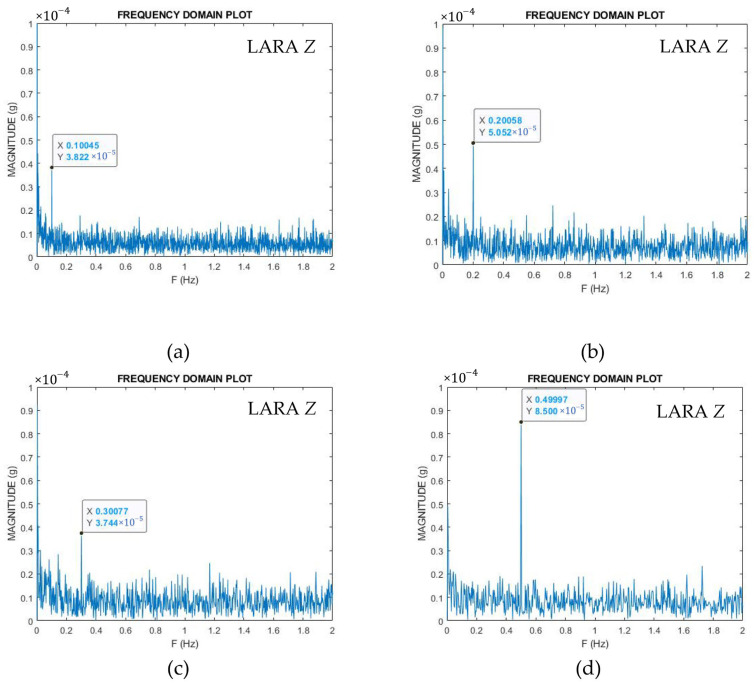
FFT representation of the low-frequency signals: (**a**) 0.1 Hz, (**b**) 0.2 Hz, (**c**) 0.3 Hz, and (**d**) 0.5 Hz.

It can be seen from Figure 4 that both CHEAP and LARA are attached to a rigid metal plate. This attachment was carried out by using an industrial adhesive (X60). X60 made by HBM company, is a two-component methyl-methacrylate adhesive that is widely used for accelerometer mounting [66]. Moreover, the metal plate containing CHEAP and LARA is bolted to the shaking table. For validating the accelerometers’ accuracy, 11 tests with various frequency ranges (from 0.1 Hz to 32 Hz) were performed. For finding the frequency report of the accelerometers, the accelerometers’ time-domain raw data were converted to the frequency domain by an FFT program written in the MATLAB software. To show the methodology of the calculation of the accelerometer’s frequency errors from the jack’s results, four frequency domain plots of LARA for 0.1 Hz (Figure 5a), 0.2 Hz (Figure 5b), 0.3 Hz (Figure 5c), and 0.5 Hz (Figure 5d) are presented in Figure 5.

The accelerometer’s frequency error is estimated by calculating the percentage error of the plot value from the reference value of the excitation device. Table 3 compares the frequency errors of LARA, CHEAP, and MPU9250 with the jack’s reference values. Table 3 is organized in columns: (1) Frequency (Hz): the reference value of the excitation device, (2) LARA’s error (%), (3) CHEAP’s error (%), and (4) MPU9250’s error (%).

Analysis of Table 3 shows that a single MPU9250 is reporting the same frequency error as the CHEAP from 2 Hz on. Since this particular MPU9250 is one of the CHEAP accelerometers, its frequency report is synchronized with CHEAP. It can also be deduced from this table that a single MPU9250 has not enough resolution for frequencies smaller than 2 Hz. Further analysis of Table 3 shows that CHEAP cannot visualize signals with a frequency smaller than 0.4 Hz either. In fact, LARA has a broader range of frequency than MPU9250 and CHEAP and can locate signals up to 0.1 Hz with 0.5% of error. In fact, the frequency range of LARA, based on the presented information, is between 0.1 Hz and its bandwidth (165 Hz).

Further investigation of Table 3 illustrates that MPU9250, CHEAP, and LARA have similar errors for signals with frequencies bigger than 2 Hz, but the error is still not 0.000%. In fact, the FFT evaluation can be influenced by irregularities, such as the number of sampled data and the sampling frequency speed [60]. However, controlling all the data for such accuracy is not the aim of this paper.

### 3.3. Acceleration Amplitude Validation

This subsection first compares the acceleration amplitude accuracy of MPU925, CHEAP, and LARA using a sine wave with a known frequency and acceleration amplitude. Then, to check the ultimate acceleration range of LARA, a laboratory experiment using a sine wave with the RMS value of 1 g was performed.

**Figure 6:**
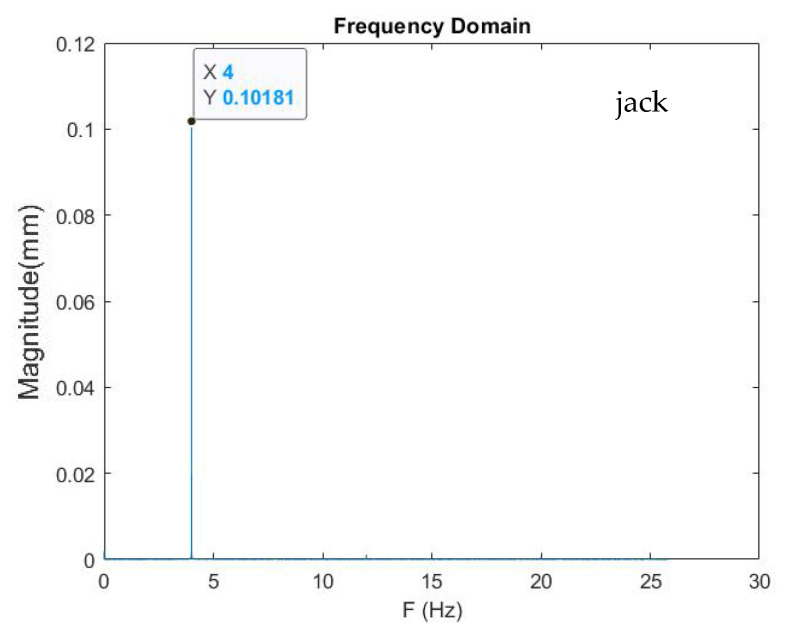
Displacement report of the jack in a frequency-domain diagram.

An experiment was carried out with the jack to compare the acceleration acquisition of MPU925, CHEAP, and LARA. The jack was calibrated to move with a frequency of 4 Hz and a displacement range of 0.1 mm from its null axis. During each test, the jack reported its time-domain information with a sampling frequency of 500 Hz. Figure 6 illustrates jack’s report for this experiment in the frequency-domain diagram.

Analyzing Figure 6 shows that the jack was moving with a frequency of 4 Hz and an averaged displacement of 0.10181 mm; consequently, the jack was working with a 1.81% error rate from 0.1 mm that the programmed displacements.

For validating the accelerometers’ accuracy, their acceleration amplitude report of the sensors was converted to displacements. Then, high-pass and low-pass filters removed signals with frequencies smaller than 0.1 Hz and bigger than 1/10 of the accelerometer’s bandwidth. These filters are MATLAB functions that can filter the signals out of the interest range.

**Figure 7:**
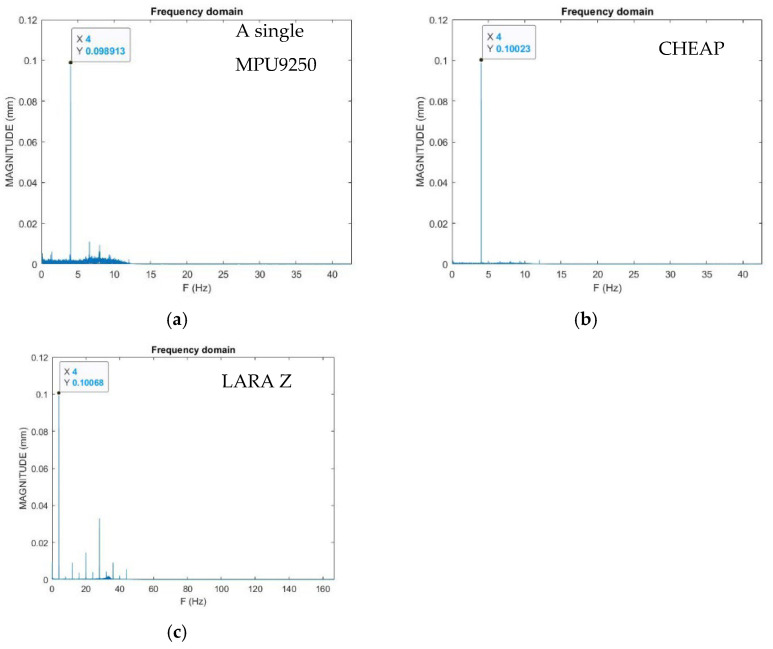
Displacement report of the accelerometers: (**a**) MPU9250, (**b**) CHEAP, and (**c**) LARA.

Finally, the FFT analysis was carried out, and obtained results are presented in Figure 7. Figure 7a–c show the reported displacement of MPU9250, CHEAP, and LARA, respectively. It should be noted that the accuracy of the accelerometers for measuring the magnitude of the induced vibration is analyzed in the frequency domain representation. It is due to the fact that making a unique noiseless signal on a shaking table or an actuator is significantly challenging [67]. In addition, for the OMA of bridges, accelerometers are mounted to measure the structural response of the bridges under ambient vibrations, such as those induced by traffic, wind, and temperature variation. Therefore, this sum of ambient vibrations is usually evaluated in the frequency domain representation [34].

Analysis of Figure 7 shows that even though all accelerometers are reporting the same frequency, they measure the induced signal’s magnitude inconsistently. MPU9250, CHEAP, and LARA have measured the jack’s displacement as 0.098913, 0.10023, and 0.10068 mm. The MPU9250’s displacement measurement is off by 1.31% from the CHEAP’s report and 2.85% off from jack’s report (Figure 6). Further analysis of Figure 7 shows that CHEAP and LARA’s magnitude report has less than 0.5% error from each other. In fact, CHEAP and LARA have measured the jack’s displacements with 1.55% and 1.2% errors from the introduced displacement measurement of the jack in Figure 6.

**Figure 8:**
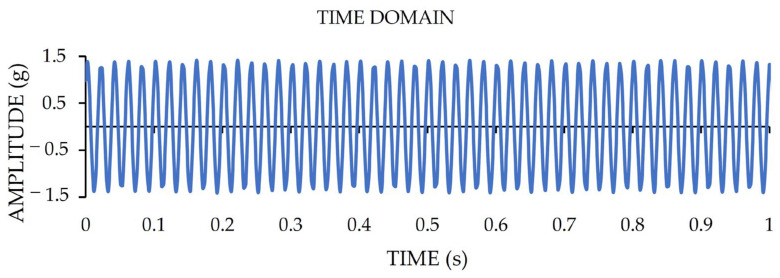
The time-domain presentation of a vibration acquisition with RMS value of one g by LARA.

It should be pointed out that the shown displacement magnitude of Figure 7c corresponds to a measured acceleration amplitude of 0.006 g. Furthermore, for measuring the top acceleration amplitude range of LARA, another test is carried out on a dynamic actuator. This time, the used dynamic actuator was programmed to induce vibrations with a Root Mean Square (RMS) value of one g. This test was performed for one minute, and, after that, the RMS value of the acquired vibrations was calculated. The time-domain representation of this test for a time duration of one second is presented in Figure 8. The calculated RMS value of the acquired data of LARA for one minute is 0.991 g. This value shows a 0.9% error from the reference RMS value of the test.

It should be noted that LARA, such as any other accelerometer, has an acceleration amplitude range for each of its axes. The sensor gets overloaded when a signal has an acceleration amplitude that exceed a certain range. It should be noted that when MEMS accelerometers reach their saturation level (get overloaded) [68], they are not able to measure the magnitude of the impact anymore [69]. In fact, LARA, unlike piezoelectric solutions, does not require a parametric analysis as it does not experience a drift after saturation [21]. More information about the saturation of MEMS accelerometers can be found in [68].

**Figure 9:**
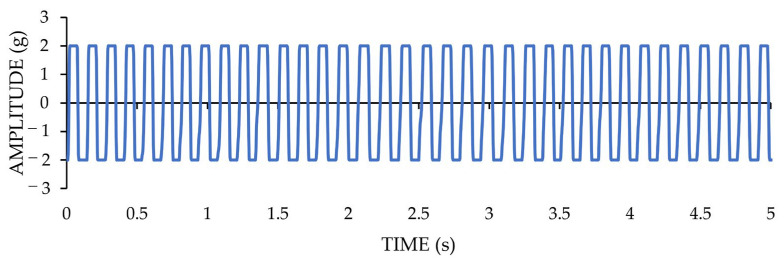
The time-domain presentation of acceleration amplitude saturation of LARA.

LARA is set to have an acceleration amplitude range of ±2 g. It should be noted that the LARA is calibrated in Applplus [70] under RMS acceleration amplitude of 0.5 g and frequency range of between 5 and 160 Hz. In addition, Figure 8 presented acceleration amplitude verification of LARA for 1 g where the maximum measured impact was 1.42 g. In order to investigate its theoretical saturation level (±2 g), an experiment with an acceleration amplitude higher than 2 g was performed. Figure 9 shows the time-domain representation of this experiment.

The analysis of Figure 9 shows that LARA could not measure the acceleration amplitude of the produced signal beyond the ±2 g magnitude as the top and bottom of the signal are cut. This means that LARA is overloaded. It is also noted in the literature that when substantial impact happens, the output voltage of MEMS accelerometers (such as LARA) reach a fixed value that does not vary (saturation) [71]. To decrease the chance of overloading of accelerometers (saturation), low-pass filters are traditionally recommended [72].

To summarize the collected information of the laboratory tests in Section 3.2 and Section 3.3, LARA accurately measured frequencies of sine waves within the range of 0.4 and 32 Hz. Furthermore, LARA accurately measured the acceleration amplitude of a sine wave with a magnitude of 0.006 g. Moreover, LARA’s maximum acceleration amplitude measurement was investigated using an induced sine wave with RMS acceleration amplitude of 1 g.

## 4. Real Structure Test and Results

This section presents a field test carried out on a short-span bridge for comparing the measured eigenfrequencies of LARA with those of a high-precision sensor.

**Figure 10:**
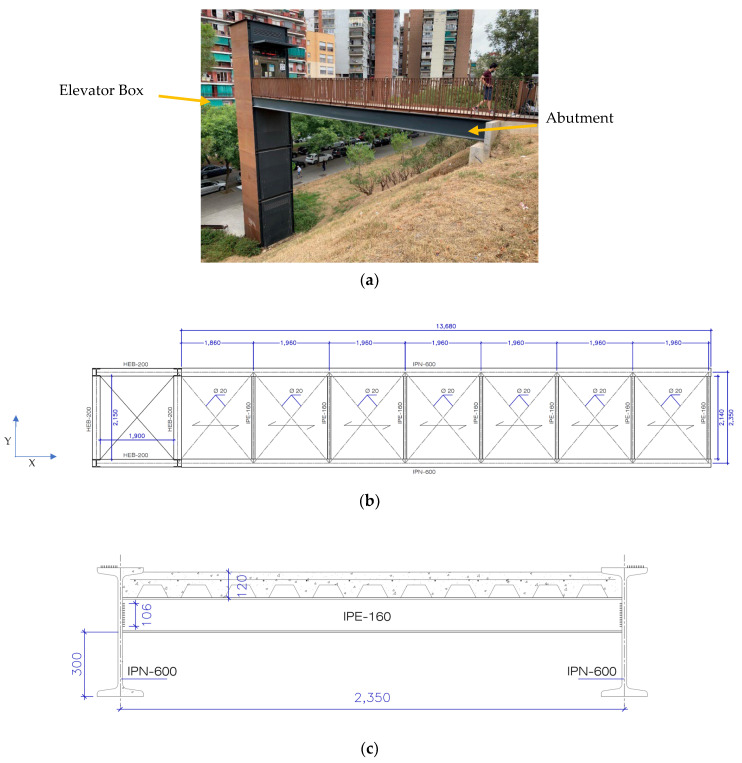
(**a**) A picture of the pass way, (**b**) plan of the bridge, and (**c**) section of the pass way bridge (all units are in mm).

In the first place, the footbridge is shown in Figure 10a. As shown, it is connected to an elevator box and from the other side, it is located on an abutment. Figure 10b,c show the plan and section of the bridge, respectively.

**Figure 11:**
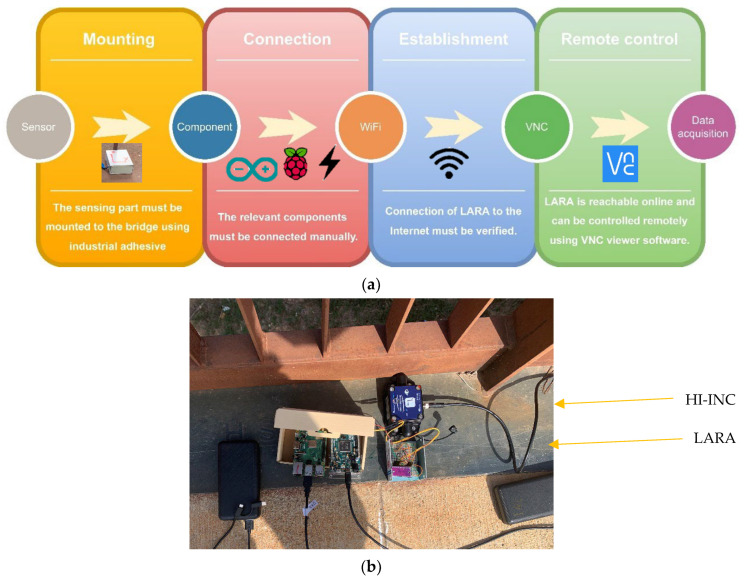
Mounting the sensors to the mid span of the bridge under study: (**a**) mounting diagram of LARA to the bridge and (**b**) photo of LARA and HI-INC sensor mounted on a footbridge in Barcelona.

After mounting the sensors to the bridge, they were connected to the rest of the monitoring components (power bank, USB 4G dongle, Arduino, and Raspberry Pi) manually. It is important to highlight that to enable its communication throughout the Internet, the Raspberry Pi was previously configured with a WiFi hotspot. In fact, after its assemblage on site the Raspberry Pi was used to initiate the data acquisition process remotely, using the Virtual Network Computing (VNC) software [73]. The acquired data was first collected on the memory card of the Raspberry Pi. Then, when the data acquisition process finished, the obtained data were moved to a computer using the VNC software. Figure 11a presents the diagram of LARA setup on the bridge. The location of the commercial dynamic sensor (HI-INC) and LARA on the mid-span of the bridge is shown in Figure 11b.

It is also important to mention that the cables shown in Figure 11b are not for connecting LARA or HI-INC to a laptop. Both systems were controlled remotely. These cables are either for power-source connection or for connecting the Arduino and the Raspberry Pi.

**Figure 12:**
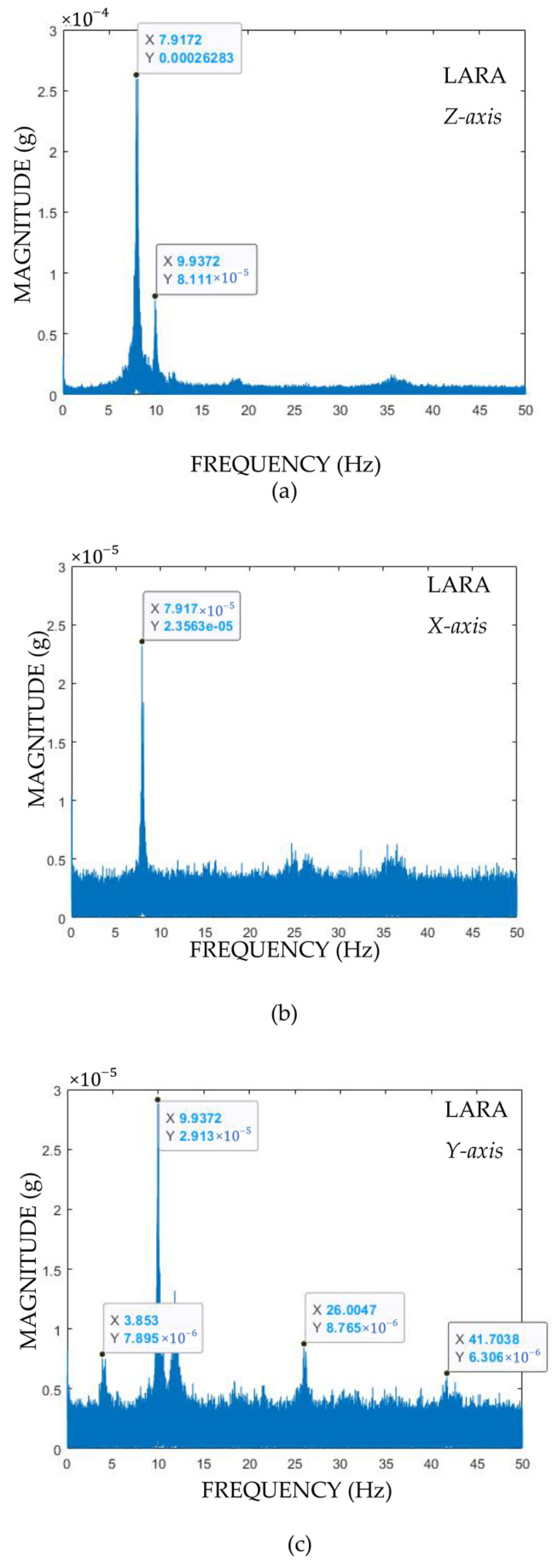
Eigenfrequency analysis of a footbridge using LARA for (**a**) vertical, (**b**) longitudinal, and (**c**) transversal directions of the footbridge.

The outputs of the eigenfrequency analysis of LARA are shown in Figure 12a–c for *x*, *y,* and *z* axis, respectively.

Analysis of Figure 12 shows that the bridge is not significantly excited about its *x*-axis (longitudinal direction). It is appeared that the bridge is excited very well about *y* and *z* axes.

Table 4 presents results of the eigenfrequency analysis of LARA and the used high-precision inclinometer dynamic (HI-INC). This table also presented the difference percentage of the measured frequencies with LARA from those of the HI-INC sensor.

Comparing the measured eigenfrequencies of LARA with those of HI-INC shows a maximum error of 1.28%.

## 5. Conclusions

Developing low-cost sensors is essential for Structural Health Monitoring (SHM) of structures with a low budget for their SHM assessments. These sensors can allow engineers to leave the equipment on the structure for monitoring. In previous works, a Cost Hyper Efficient Arduino Product (CHEAP) was introduced, and its performance was validated in laboratory experiments. This device was a uniaxial accelerometer with a sampling frequency of 85 Hz. The main novelty of CHEAP was to improve the resolution, accuracy, and noise level by using the averaged result of five low-cost digital accelerometers located on the same spot for sampling the same signal from the same direction. However, CHEAP presented a few drawbacks such as a low sampling frequency, not being independent of a computer, being uniaxial, being heavy, having a high Noise Density (ND), not having access to the time of the Internet for future sensor synchronizations, and not being able to be controlled entirely wireless. By upgrading both hardware and software of the CHEAP, a new triaxial Low-cost Adaptable Reliable Accelerometer (LARA) is introduced. The advantages of LARA with respect to CHEAP are:

(1) By rewriting codes, LARA achieved a faster sampling frequency;
(2) Having a connected Raspberry Pi to the Internet made LARA independent of any attached computer for data sampling;
(3) Having aligned correctly, its accelerometers made LARA triaxial;
(4) Having a better and more efficient connection reduced LARA’s ND;
(5) Having the time of the Internet for future data synchronization;
(6) Being completely wireless excluded the need for an engineer’s presence on the site for data acquisition.

To evaluate the LARA performance, resolution, and ND, a set of laboratory experiments was performed. These obtained results show that LARA has less ND ratio than MPU9250 and CHEAP for the same number of acquired data. Furthermore, comparing the effects of MPU9250, CHEAP, and LARA with the reports of the shaking table show that LARA is more accurate in reporting both frequency and acceleration amplitude than MPU9250 and CHEAP in a laboratory experiment with the same time duration.

A field test on a footbridge in Barcelona with a span length of 14 m has been carried out using LARA accelerometer. The generated frequencies of the eigenfrequency analysis from LARA have been validated with a commercial high-precision sensor (HI-INC).

## Figures and Tables

**Table 2:** Comparison of commercial triaxial MEMS accelerometers with LARA.

No.	Name	Acceleration Range (G)	Sampling Frequency (Hz)	Spectral Noise (µg/√Hz)	Sensitivity (V/G)	Price (€)	Acquisition System
1	IAC-Hires [61]	±2.0	800	8	2.000	1192	Recovib Monitor
2	3713F112G [62]	±2.0	500	10	0.675	2130	482C27
3	Unquake [63]	±2.0	500	25	0.400	2500	Independent
4	Recovib Tiny [64]	±2.0	500	30	0.600	1125	Independent
5	LARA	±2.0	333	51	0.625	* 140	Independent
6	CHEAP	±2.0	85	162	0.625	^†^ 84	A computer
7	MPU9250	±2.0	85	390	0.625	^‡^ 50	A computer

* Research prototype; ^†^ Research prototype; and ^‡^ Research prototype.

**Table 3:** Frequency validation of the accelerometers.

Frequency (Hz)	LARA’s Error (%)	CHEAP’s Error (%)	MPU9250’s Error (%)
0.1	0.450		
0.2	0.295		
0.3	0.260		
0.4	0.050	0.470	
0.5	0.006	0.011	
2	0.005	0.025	0.025
4	0.000	0.000	0.000
8	0.003	0.003	0.003
10	0.004	0.004	0.004
16	0.001	0.001	0.001
32	0.003	0.003	0.003

**Table 4:** Comparison of the first three mode steps frequencies of LARA with HI-INC.

Mode Number	HI-INC	LARA	Difference
1	3.90 Hz	3.85 Hz	1.28%
2	7.91 Hz	7.91 Hz	0.20%
3	9.94 Hz	9.93 Hz	0.88%
4	26.25 Hz	26.01 Hz	0.91%
5	42.81 Hz	41.70 Hz	0.27%

## Data Availability

Not applicable.
